# Susceptibility of Measles Virus to World Health Organization–Recommended Hand Rubs, Oral Rinses, and Surface Disinfectants

**DOI:** 10.1093/ofid/ofaf627

**Published:** 2025-10-14

**Authors:** Lukas Daniel Sandoval Flores, Marylyn Martina Addo, Eike Steinmann, Toni Luise Meister

**Affiliations:** Institute for Infection Research and Vaccine Development, Centre for Internal Medicine, University Medical Centre Hamburg-Eppendorf, Hamburg, Germany; Department for Clinical Immunology of Infectious Diseases, Bernhard Nocht Institute for Tropical Medicine, Hamburg, Germany; German Centre for Infection Research, Partner Site Hamburg-Lübeck-Borstel-Riems, Hamburg, Germany; Institute for Infection Research and Vaccine Development, Centre for Internal Medicine, University Medical Centre Hamburg-Eppendorf, Hamburg, Germany; Department for Clinical Immunology of Infectious Diseases, Bernhard Nocht Institute for Tropical Medicine, Hamburg, Germany; German Centre for Infection Research, Partner Site Hamburg-Lübeck-Borstel-Riems, Hamburg, Germany; Department for Molecular and Medical Virology, Ruhr University Bochum, Bochum, Germany; German Centre for Infection Research, External Partner Site Bochum, Bochum, Germany; Institute for Infection Research and Vaccine Development, Centre for Internal Medicine, University Medical Centre Hamburg-Eppendorf, Hamburg, Germany; Department for Clinical Immunology of Infectious Diseases, Bernhard Nocht Institute for Tropical Medicine, Hamburg, Germany; German Centre for Infection Research, Partner Site Hamburg-Lübeck-Borstel-Riems, Hamburg, Germany

**Keywords:** measles virus, stainless steel, surface disinfection, surface stability, World Health Organization–recommended hand rub formulations I and II

## Abstract

**Background:**

Measles continues to pose a major public health threat worldwide. Reoccurring outbreaks are fueled by declining vaccination rates, increased global travel, migration, and disruptions in immunization programs. Consequently, hygiene measures remain essential to prevent transmission, particularly in health care settings where nosocomial infections can occur.

**Methods and Results:**

To address this, we tested virus inactivation by common oral rinses, as primary transmission occurs via aerosols and droplets, using a quantitative suspension assay in accordance with European guidelines. We further evaluated World Health Organization–recommended hand rub formulations based on ethanol and 2-propanol with the same assay. After confirming that measles virus remains viable for several hours on stainless steel, we assessed the efficacy of surface disinfectants, including alcohol-, aldehyde-, and hydrogen peroxide–based products.

**Conclusions:**

The virus was effectively inactivated by World Health Organization–recommended hand rub formulations, oral rinses, and surface disinfectants, demonstrating the effectiveness of standard hygiene measures in infection control. These findings underscore the critical importance of consistent hygiene practices in limiting measles transmission.

Measles continues to be a major global health issue, with current outbreaks and increasing numbers of cases reported in multiple regions. It is a highly contagious viral disease caused by measles virus (MeV), determined as one of the leading causes of vaccine-preventable mortality among young children worldwide. In 2023, approximately 10.3 million measles cases and >107 500 deaths were recorded, with the greatest impact seen in low-income countries lacking adequate health care infrastructure [1]. Infants between 6 and 11 months of age are especially vulnerable, facing a heightened risk of severe complications and death [2]. In recent years, measles cases have resurged, driven by declining vaccination rates, increased global travel, migration, and disruptions in immunization programs—factors exacerbated by the COVID-19 pandemic and international conflicts [1, 3].

While many cases are mild and self-limiting, serious complications such as pneumonia and debilitating encephalitis can occur, particularly in young children, pregnant individuals, and patients who are immunocompromised [2]. Due to its high transmissibility through respiratory droplets and aerosols, measles presents a considerable threat in health care environments, where unprotected staff can contribute to nosocomial transmission [1, 3]. MeV RNA has indeed been detected in environmental samples, including air specimens and surface swabs collected from the hospital room of an infected patient [4]. Consequently, several reports have identified health care workers as index cases in hospital outbreaks, particularly when unvaccinated, thereby contributing to the spread of the virus within these settings [5, 6].

Therefore, effective infection control measures are crucial to limiting the spread of MeV [2, 3, 7]. But data on the environmental stability of MeV and its susceptibility to disinfectants remain limited [2]. Thus, this study investigates disinfection profiles and the virus's stability on stainless steel, chosen for its relevance in hospital environments and because it serves as the reference surface in European guidelines. The objective is to inform risk assessments aimed at minimizing transmission of MeV in hospital settings.

## METHODS

### Cell Culture and Virus Propagation

Vero E6 cells were cultured in Dulbecco's modified Eagle's medium supplemented with 10% (v/v) fetal calf serum (FCS), 1% (v/v) nonessential amino acids, 100-IU/mL penicillin, 100-mg/mL streptomycin, and 2-mmol/L L-glutamine. For MeV production (ATCC VR-24), Vero E6 cells were seeded at 3 × 10^6^ cells/flask. After 24 hours, the cells were inoculated with MeV (multiplicity of infection, 0.03) and incubated for 72 hours at 37 °C with 5% CO_2_. Upon visual cytopathic effect, the cells were scraped in Opti-MEM (Gibco) and particles released by 1 freeze-thaw cycle. The virus suspension was cleared from cell debris by centrifugation (1000 × *g* for 5 minutes) and stored at −80 °C until further usage. Infectious viral titers were determined by an endpoint dilution assay.

### Quantitative Suspension Test

We set out to determine the virucidal activity of 7 oral rinses (Table 1) and the inactivation capacity of World Health Organization (WHO)–recommended hand rub formulations I and II (Table 2), as well as their active ingredients ethanol and 2-propanol. Assessment was based on European guideline EN14476 as described previously [8]. In brief, 8 parts of disinfectant per oral rinse or cell culture medium for the untreated control were mixed with 1 part of interfering substance (bovine serum albumin; final concentration, 0.3 g/L; clean condition) and 1 part of MeV and incubated for 30 seconds at room temperature. WHO formulations I and II, as well as ethanol and 2-propanol, were tested for final concentrations of 20%, 30%, 40%, 60%, and 80%. Oral rinses were tested for a final concentration of 80%. An endpoint dilution assay was performed on Vero E6 cells to determine the remaining infectious viral titers. After 7 days, cytopathic effects were evaluated microscopically and used to calculate the 50% tissue culture infectious dose per milliliter (TCID_50_/mL).

**Table 1.** Composition of Commercially Available Oral Rinses

Product^a^	Ingredients	Product Concentration, %	Incubation Time, s
Chlorhexamed FORTE	0.2% (w/v) chlorhexidine bis(D-gluconate), peppermint flavor, macrogolglycerol hydroxystearate, glycerol, sorbitol solution 70%, water	80	30
Listerine–Cool Mint (Mild)	Aqua, propylene glycol, sorbitol, poloxamer 407, sodium lauryl sulfate, eucalyptol, benzoic acid, sodium benzoate, methyl salicylate, thymol, sodium saccharin, sodium fluoride (220 ppm), menthol, sucralose, aroma, CI 42053	80	30
Listerine–Total Care	Aqua, alcohol, sorbitol, poloxamer 407, benzoic acid, sodium saccharin, eucalyptol, methyl salicylate, thymol, aroma, menthol, sodium benzoate, CI 47005, CI 42053	80	30
Meridol Gum Protection	Aqua, glycerin, xylitol, PVP, oleaminopropylamineth-3 HCl, zinc lactate, polyglyceryl-4 caprate, aroma, sodium fluoride, sodium citrate, saccharin, sucralose, CI 42051, natriumfluorid (250 ppm)	80	30
Dontodent Junior (>6 y)	Aqua, glycerin, sorbitol, disodium phosphate, propylene glycol, allantoin, chamomilla recutita flower extract, xylitol, olaflur, sodium fluoride (250 ppm), sodium saccharin, citric acid, aroma, sodium benzoate, potassium sorbate	80	30
Odol-med3 Junior (>6 y)	Aqua, glycerin, sorbitol, poloxamer 338, PEG-60 hydrogenated castor oil, aroma, allantoin, cetylpyridinium chloride, methylparaben, sodium benzoate, sodium fluoride (225 ppm), sodium saccharin, disodium phosphate, sodium phosphate, limonene, CI 42051	80	30
Listerine–Smart Kidz (>6 y)	Aqua, sorbitol, aroma, phosphoric acid, sucralose, cetylpyridinium chloride, sodium fluoride (100 ppm), menthol, disodium phosphate, benzyl alcohol, CI 16035, CI 42053	80	30

Abbreviation: ppm, parts per million.

^a^The exact formulations for these oral rinses are not publicly available due to patent-related restrictions.

**Table 2.** Composition of WHO-Recommended Hand Rub Formulations

Product	Ingredients	Product Concentration, %	Incubation Time, s
WHO formulation I	80% ethanol (v/v) 1.45% glycerol (v/v) 0.125% hydrogen peroxide (v/v)	20, 30, 40, 60, 80	30
WHO formulation II	75% 2-propanol (v/v) 1.45% glycerol (v/v) 0.125% hydrogen peroxide (v/v)	20, 30, 40, 60, 80	30

Abbreviation: WHO, World Health Organization.

### MeV Stability Testing

Stainless-steel discs (2-cm diameter discs, article 4174-3000; GK Formblech GmbH) were decontaminated in 70% (v/v) ethanol for 15 minutes. The discs were subsequently contaminated with 50 µL of virus solution containing 9 parts of MeV and 1 part of interfering substance (bovine serum albumin; final concentration, 0.3 g/L; clean condition). All specimens were stored at room temperature. Virus was recovered at time points indicated in the figure legend postcontamination by transferring the specimens into a 25-mL container harboring 2 mL of cell culture medium (without FCS) and subsequent vortexing. For each time point, 3 specimens were collected. An endpoint dilution assay was performed on Vero E6 cells as described previously. Humidity (39.7% ± 3.4%, mean ± SD) and temperature (26.2°C ± 1.3°C) were simultaneously measured over the course of the experiment.

### MeV Inactivation by Surface Disinfectants

Stainless-steel discs were decontaminated and spiked with virus solution as described previously. The steel discs were incubated until the virus solution was desiccated completely. Subsequently, 100 µL of surface disinfectant (Table 3) at indicated concentrations was applied onto the carrier and incubated according to the manufacturer's instructions. Cell culture medium was used for the untreated control. Thereafter, the specimens were transferred into a 25-mL container harboring 2 mL of cell culture medium (without FCS), and virus was recovered by subsequent vortexing. An endpoint dilution assay was performed on Vero E6 cells as described earlier.

**Table 3.** Composition of Surface Disinfectants

Product	Ingredients	Product Concentration, %	Incubation Time
Bacillol AF	450 mg/g, 1-propanol 250 mg/g, 2-propanol 47 mg/g, ethanol	80	30 s
Antifect N liquid	250 mg/g, ethanol 350 mg/g, 2-propanol	80	30 s
Kohrsolin FF	50 mg/g, glutaraldehyde 30 mg/g, benzylalkyldimethyl-ammonium chloride 30 mg/g, didecyldimethyl-ammonium chloride	0.5	5 min
Incidin Rapid	98 mg/g, glutaraldehyde 50 mg/g, alkyldimethylbenzyl-ammonium chloride 50 mg/g, didecyldimethyl-ammonium chloride	0.5	5 min
Incidin OxyFoam	15 mg/g, hydrogen peroxide	80	30 s

### Statistical Analysis

Inactivation kinetics of MeV by WHO formulations I and II was compared with other respiratory viruses by using a robust Hill nonlinear dose-response fit. Thereby, comparing respiratory viruses included influenza virus H1N1, bovine coronavirus, severe acute respiratory syndrome coronavirus (SARS-CoV), Middle East respiratory syndrome coronavirus (MERS-CoV), and SARS-CoV-2, as well as the reference virus modified vaccinia Ankara.

## RESULTS

### Inactivation of MeV by Commercially Available Oral Rinses

**Figure 1.**
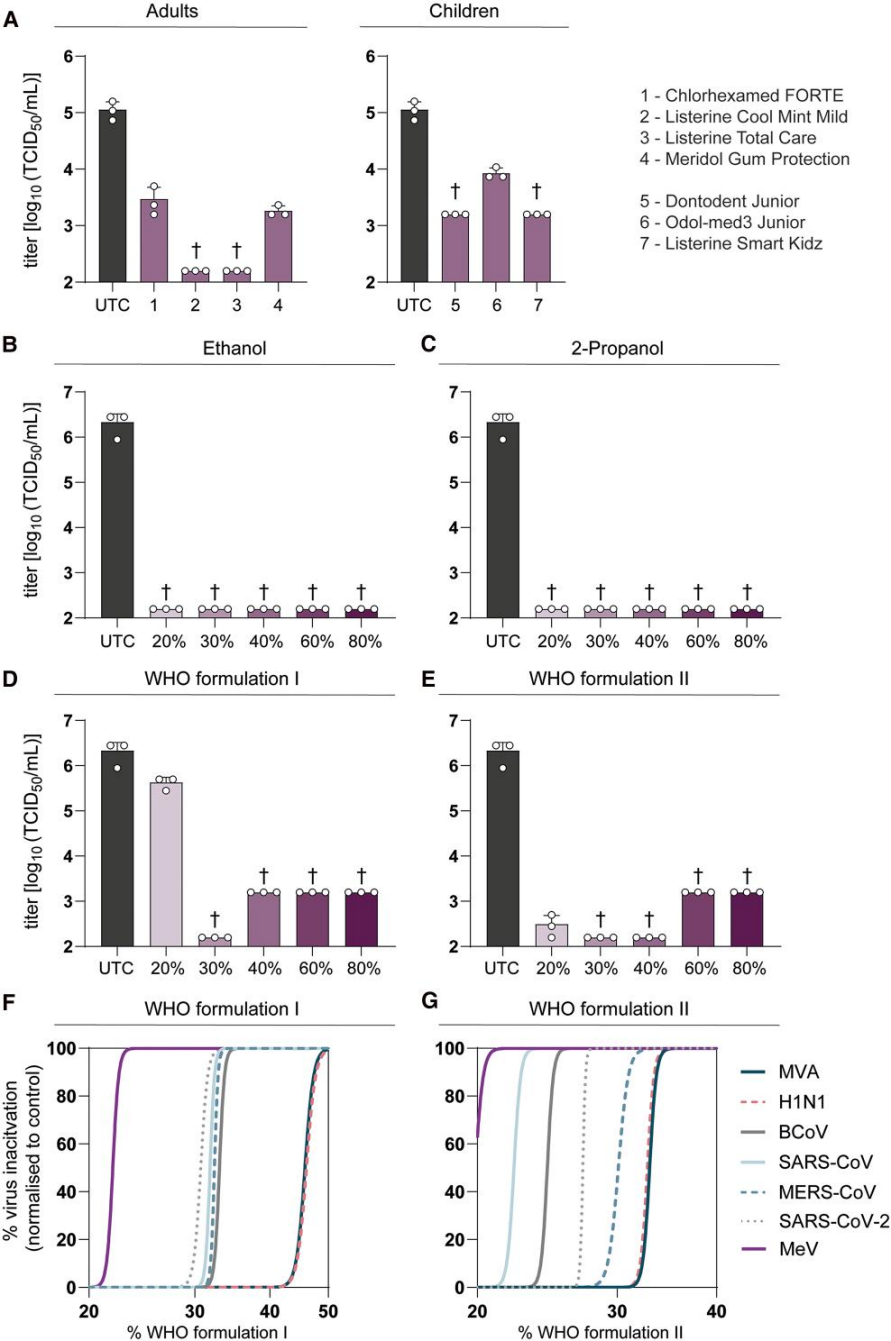
Inactivation of MeV by oral rinses, WHO-recommended hand rub formulations, and their active ingredients ethanol or 2-propanol. All oral rinses and disinfectants were tested regarding their potential to inactivate MeV in a quantitative suspension test according to EN14476. *A*, Eight-part oral rinses were mixed with 1-part interfering substance (bovine serum albumin; final concentration, 0.3 g/L) and 1-part MeV for 30 seconds. Remaining infectious viral titers were determined in an endpoint dilution assay and are displayed as 50% tissue culture infectious dose per milliliter (TCID_50_/mL). The untreated control (UTC) is displayed as the dark gray bar. The purple bars show viral titers recovered after exposure to the oral rinses. Ethanol and 2-propanol (*B* and *C*) as well as WHO formulations I and II (*D* and *E*) were diluted to 20%, 30%, 40%, 60%, and 80% final concentration and mixed with 1-part interfering substance (bovine serum albumin; final concentration, 0.3 g/L) and 1-part MeV for 30 seconds. Remaining infectious viral titers were determined in an endpoint dilution assay and are displayed as TCID_50_/mL. The UTC is displayed as the dark gray bar. The purple bars show viral titers recovered after exposure to the disinfectants. The cross (†) indicates a reduction of infectious viral titers to the lower limit of detection. Data are presented as mean ± SD. *F* and *G*, Inactivation kinetics of MeV by WHO-recommended hand rub formulations I and II were compared with other respiratory viruses by using a robust Hill nonlinear dose-response fit. Respiratory viruses included influenza virus H1N1, bovine coronavirus, severe acute respiratory syndrome coronavirus (SARS-CoV), Middle East respiratory syndrome coronavirus (MERS-CoV), and SARS-CoV-2, as well as the reference virus modified vaccinia Ankara (MVA). BCoV, bovine coronavirus; MeV, measles virus; WHO, World Health Organization.

MeV is predominantly transmitted by respiratory droplets; thus, oral rinses may possess the ability to reduce the risk of transmission by temporarily lowering the viral load in the oral cavity. Therefore, we tested the virucidal efficacy of commercially available oral rinses for adults and children (aged >6 years) according to EN14476 (Figure 1*A*, Table 1). All tested oral rinses significantly reduced viral loads; however, residual infectious virus was still detectable following exposure to Chlorhexamed FORTE (reduction factor, 1.58 log_10_ TCID_50_/mL), Meridol Gum Protection (1.79 log_10_ TCID_50_/mL), and Odol-med3 Junior (1.12 log_10_ TCID_50_/mL). In contrast, all 3 Listerine products and Dontodent Junior decreased viral titers to the lower limit of quantification (LLOQ), suggesting efficient inactivation of MeV with applicable oral rinses.

### Inactivation of MeV by WHO-Recommended Hand Rub Formulations and Their Active Ingredients Ethanol and 2-Propanol

By employing a quantitative suspension test according to EN14476, the virucidal activity of different concentrations (20%, 30%, 40%, 60%, 80%) of ethanol and 2-propanol was evaluated. Therefore, 8 parts of alcohol were mixed with 1 part of interfering substance to mimic protein contamination and 1 part of virus. Both alcohols at a 20% concentration were effective in reducing infectious viral loads to the LLOQ (Figure 1*B* and 1*C*). WHO-recommended hand rub formulations I and II were subsequently tested in a similar approach (Table 2). In both cases, 30% was sufficient to completely inactivate MeV. Formulation I at 20% only slightly reduced MeV titers (0.71 log_10_ TCID_50_/mL). In contrast, formulation II at 20% reduced infectious titers by >3.5 log_10_ TCID_50_/mL, although some infectious virus remained detectable (Figure 1*D* and 1*E*). A comparison of the inactivation kinetics of WHO-recommended hand rub formulations I and II with those of other respiratory viruses—including influenza A virus H1N1, bovine coronavirus, SARS-CoV, SARS-CoV-2, and MERS-CoV—revealed that MeV exhibited the greatest susceptibility to inactivation by alcohol-based hand rubs (Figure 1*F* and 1*G*).

### Environmental Stability of MeV

**Figure 2.**
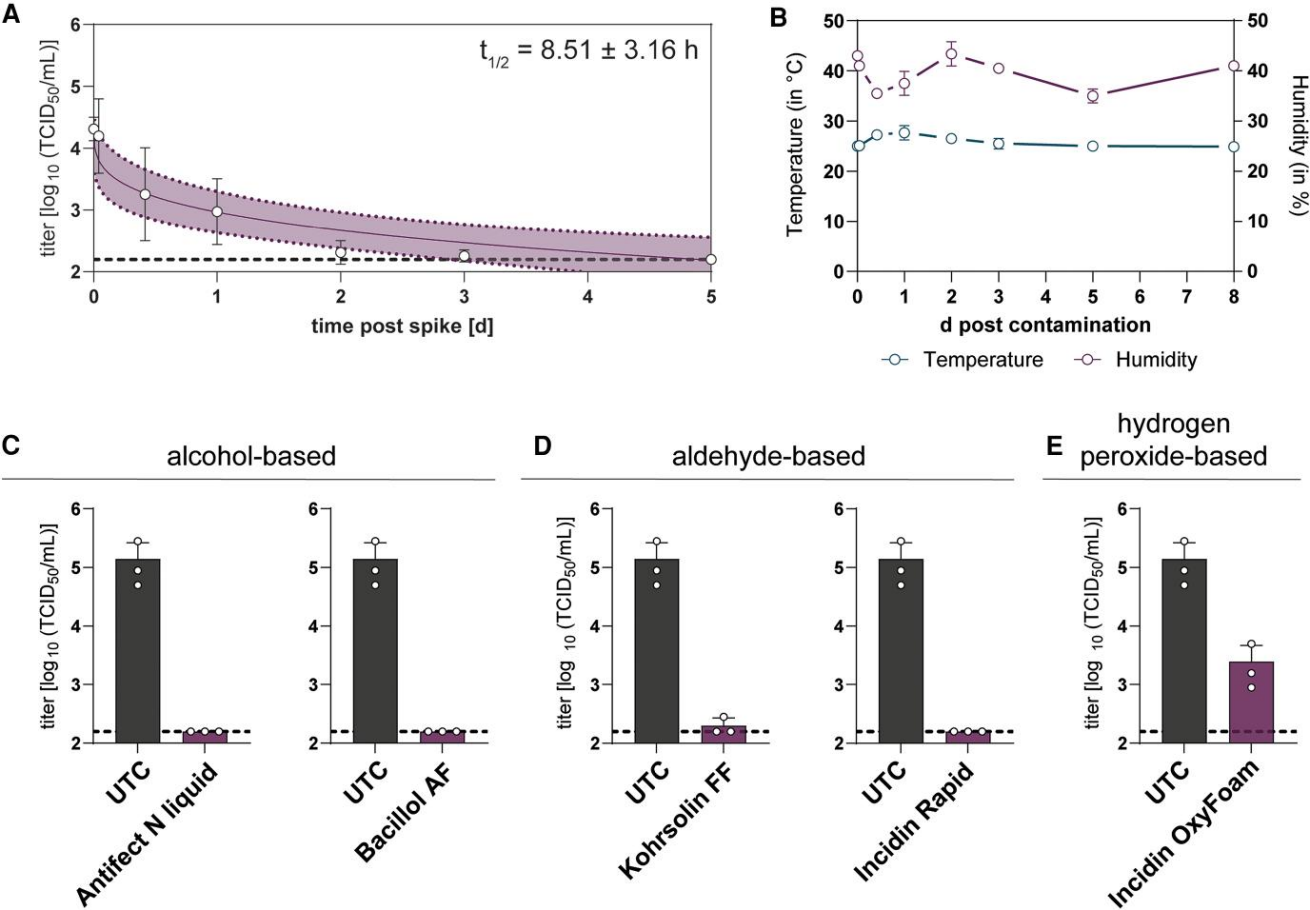
Stability of measles virus (MeV) on stainless-steel disc and inactivation by surface disinfectants. *A*, Environmental stability of MeV on stainless-steel discs. Stainless-steel discs were contaminated with 50-µL virus suspension (45-µL MeV mixed with 5-µL bovine serum albumin; final concentration, 0.3 g/L) and incubated for the indicated periods. Virus was recovered from the discs, and remaining infectious viral titers were determined in an endpoint dilution assay and are displayed as 50% tissue culture infectious dose per milliliter (TCID_50_/mL). A nonlinear model based on Weibull distribution was used to fit data. *B*, Simultaneously, environmental data (temperature and humidity) was collected at each day. Five surface disinfectants based on (*C*) alcohol, (*D*) aldehyde, and (*E*) hydrogen peroxide were tested according to EN16777 to evaluate their virucidal activity. All products were tested as suggested by the manufacturers. Nine-part MeV mixed with 1-part interfering substance (bovine serum albumin, 0.3 g/L; final concentration) were spiked on sterile stainless-steel discs and inactivated with undiluted Bacillol, Antifect liquid, and Incidin OxyFoam for 30 seconds. Kohrsolin and Incidin Rapid were diluted to 0.5% working solutions and incubated for 5 minutes on contaminated stainless-steel discs. Remaining infectious viral titers were determined in an endpoint dilution assay and are displayed as TCID_50_/mL. The untreated control (UTC) is displayed as the dark gray bar. The purple bars show viral titers recovered after exposure to the surface disinfectants. The dotted line indicates the lower limit of detection. Data are presented as mean ± SD.

The duration that viruses remain infectious on inanimate surfaces varies by their intrinsic properties and surrounding environmental conditions. To evaluate the environmental stability of MeV, stainless-steel discs were contaminated with infectious MeV, and remaining infectious virus was retrieved from the specimens at different time points. The evaluation of viral stability revealed the presence of infectious virus for 3 days (182.67 TCID_50_/mL). However, MeV titers dropped significantly within 1 day (1377.33 TCID_50_/mL), and almost no infectious virus was detectable after 2 days (219 TCID_50_/mL), resulting in a half-life of 8.51 ± 3.16 hours (Figure 2*A*). During the experiment, temperature and humidity were kept relatively stable at 26.2 ± 1.3 °C and 39.7% ± 3.4% (Figure 2*B*). These data suggest that even though the half-life of MeV is comparably short, transmission via inanimate surfaces can pose a risk of infection.

### Inactivation of MeV by Surface Disinfectants

To reduce the risk of fomite-mediated transmission, the application of surface disinfectants serves as an effective preventive measure. In this study, we evaluated 5 disinfectants for their ability to inactivate MeV. These included alcohol-based formulations (Antifect N liquid and Bacillol AF), aldehyde-based disinfectants (Kohrsolin FF and Incidin Rapid), and a hydrogen peroxide–based product (Incidin OxyFoam; Figure 2*C*–*E*, Table 3). The experiment was performed according to European guideline EN16777. We found that all disinfectants but Incidin OxyFoam reduced infectious viral titers to the LLOQ (158 TCID_50_/mL) within the exposure time and concentration implicated by the manufacturers. The hydrogen peroxide–based disinfectant reduced viral titers but did not achieve complete inactivation of MeV. In conclusion, depending on the active ingredients MeV can efficiently be inactivated by surface disinfectants.

## DISCUSSION

In this study, we conducted a comprehensive investigation into the environmental stability of MeV and its vulnerability to various chemical disinfectants. Our objective was to generate data that provide evidence-based guidance to reduce the risk of nosocomial transmission.

Reducing viral load in the oral cavity could lower the risk of MeV transmission via respiratory droplets. In line with this concept, our evaluation of commercially available oral rinses for adults and children (aged >6 years) demonstrated effective inactivation of MeV according to EN14476 (Figure 1*A*). Similarly, other enveloped viruses, such as SARS-CoV-2 and respiratory syncytial virus, have been efficiently inactivated by a range of oral rinses [8, 9]. However, this efficacy was directly affected by the type of active ingredient used and their concentration [10]. For instance, ingredients such as chlorhexidine, essential oils, povidone-iodine, benzalkonium chloride, cetylpyridinium chloride, octenidine dihydrochloride, and various surfactants have been shown to inactivate SARS-CoV-2 in a dose-dependent manner, whereas hydrogen peroxide and dequalinium chloride failed to have the same effect [10].

We further demonstrated that ethanol and 2-propanol, even at concentrations as low as 20%, were capable of reducing infectious MeV titers to the LLOQ (Figure 1*B* and 1*C*). These alcohols are the primary active components of WHO-recommended hand rub formulations I and II, both of which effectively inactivated MeV at a 30% concentration (Figure 1*D* and 1*E*). Recent studies, including our own, have shown that these formulations and their constituent alcohols also effectively inactivate a range of enveloped viruses, including SARS-CoV-2, MERS-CoV, SARS-CoV, mpox virus, and others [11, 12]. Notably, among the respiratory viruses tested, MeV exhibited the highest sensitivity to inactivation by WHO formulations I and II (Figure 1*F* and 1*G*). Overall, WHO formulation II (based on 2-propanol) inactivated enveloped viruses more efficiently than formulation I (based on ethanol), likely because 2-propanol exhibits lower interfacial tension and its molecular structure may allow for more effective disruption of lipid bilayers [13].

Depending on viral characteristics and environmental factors, viruses remain infectious on inanimate surfaces for a certain period. For example, SARS-CoV-2 was found to remain infectious on stainless-steel discs for 5 days, whereas even longer stability was observed for hepatitis E virus, hepatitis C virus, hepatitis A virus, and mpox virus. In comparison with these viruses, MeV appeared to be less stable on stainless steel at 3 days (Figure 2*A*). Nonetheless, MeV RNA was identified in air specimens, on environmental surfaces, and on respirators within the hospital setting of a patient who was infected [4], indicating that MeV-contaminated fomites may indeed be present in health care environments. This underlines the potential risk of environmental persistence and highlights the importance of effective surface disinfection as a preventive measure.

To mitigate this risk, the use of surface disinfectants can be an effective intervention. In this study, 5 surface disinfectants based on alcohol (Antifect N liquid and Bacillol AF), aldehyde (Kohrsolin FF and Incidin Rapid), and hydrogen peroxide (Incidin OxyFoam) were evaluated regarding their potential to inactivate MeV (Figure 2*B*–*D*). All disinfectants tested, with the exception of Incidin OxyFoam, reduced infectious MeV titers to the LLOQ within the manufacturer-recommended exposure time and concentration. This observation aligns with previous findings showing that hydrogen peroxide–based disinfectants also failed to fully inactivate viruses such as yellow fever virus and mpox virus [14, 15], suggesting that concentration and exposure time affect inactivation, particularly if the product is formulated as a foam or gel, which could limit direct contact with the viral particles [16].

## CONCLUSION

Differences in viral inactivation efficacy underscore the need for tailored hygiene measures, as they cannot be universally applied. While in vitro results provide valuable insight, it is important to recognize that these experiments are conducted under controlled conditions optimized for virus survival. Real-world factors such as fluctuating temperatures, humidity, and the presence of other microorganisms can significantly influence viral persistence, meaning that laboratory results may not fully reflect practical scenarios. Nonetheless, the demonstrated effectiveness of alcohols, WHO formulations, and surface disinfectants against MeV in laboratory conditions supports their use in health care environments and outbreak responses to help prevent transmission, while highlighting the need to consider environmental complexities when interpreting these findings.
